# Personality traits, mindfulness, and perceived stress in Chinese adults: a sequential explanatory mixed-methods approach

**DOI:** 10.3389/fpsyg.2024.1498458

**Published:** 2025-01-03

**Authors:** Litang Zhao

**Affiliations:** Faculty of Public Administration, Guizhou University of Finance and Economics, Guiyang, China

**Keywords:** perceived stress, personality traits, Big Five, mindfulness, mindfulness-attention, mindfulness-acceptance, mixed-methods, Chinese adults

## Abstract

**Background:**

This study explores how personality traits and mindfulness facets interact to influence perceived stress, focusing on a Chinese adult sample. It aims to address gaps in understanding the combined effects of dispositional and mindfulness factors on stress.

**Methods:**

A sequential explanatory mixed-methods design was employed. In the quantitative phase, 637 Chinese adults completed surveys measuring personality traits, mindfulness (attention, acceptance), and perceived stress. Hierarchical multiple regression, moderation, and mediation analyses were conducted. In the qualitative phase, semi-structured interviews with selected participants provided deeper insights into the quantitative findings.

**Results:**

Neuroticism (*β* = 0.29, *p* < 0.001) and conscientiousness (*β* = 0.15, *p* < 0.01) were positively associated with perceived stress, while mindfulness-acceptance (*β* = −0.25, *p* < 0.001) was a significant negative predictor. Neuroticism and mindfulness-acceptance uniquely explained 8 and 6% of the variance in stress, respectively. Mindfulness-attention moderated the relationship between agreeableness and stress, amplifying agreeableness’ stress-buffering effect in individuals with low mindfulness-attention. Mediation analysis revealed mindfulness-acceptance partially mediated the agreeableness-stress link. Qualitative interviews underscored the role of personality and mindfulness in shaping stress responses and coping mechanisms.

**Conclusion:**

The findings highlight mindfulness-acceptance as a critical factor in reducing stress, particularly in individuals with agreeable personalities. These results support the development of mindfulness-based interventions targeting acceptance to enhance stress resilience across diverse personality profiles.

## Introduction

Stress significantly affects mental and physical health, contributing to various psychological disorders and chronic illnesses (Cohen et al., 2007; Schneiderman et al., 2005; Shah et al., 2021). Understanding the factors that influence how people perceive and cope with stress is essential for developing effective interventions.

The Five-Factor Model (FFM) of personality is a widely recognized framework for understanding individual differences, including neuroticism, extraversion, agreeableness, conscientiousness, and openness to experience (Allen et al., 2017; Costa and McCrae, 1992; John and Srivastava, 1999). Neuroticism, marked by emotional instability and a tendency toward negative emotions, is consistently linked to higher perceived stress and maladaptive coping strategies, such as avoidance and rumination (Kendler et al., 2006; Lahey, 2009; Ormel et al., 2013). In contrast, conscientiousness and agreeableness are often associated with lower perceived stress and more effective coping, including proactive problem-solving and seeking social support (Afshar et al., 2015; Graziano and Eisenberg, 1997; Roberts et al., 2009). However, the impact of these traits on stress perception can vary depending on the context, indicating a need for more in-depth investigation (Luo et al., 2023; Piekarska, 2020).

Mindfulness, originating from Buddhist practices, has gained attention in psychological research for its role in improving mental health (Keng et al., 2011; Murphy, 2016). It is defined as paying attention to the present moment with a non-judgmental attitude and includes facets such as mindfulness-attention and mindfulness-acceptance (Baer et al., 2006; Kabat-Zinn, 2003). Mindfulness practices can reduce stress and enhance emotional regulation by increasing awareness and acceptance of present experiences (Garland et al., 2017; Green and Kinchen, 2021; Grossman et al., 2004; Strohmaier et al., 2021). Specifically, mindfulness-acceptance allows individuals to engage with their thoughts and emotions in a non-reactive way, which can help mitigate stress (Hölzel et al., 2011).

Despite extensive research on personality traits, mindfulness, and stress, there is limited understanding of their combined effects. Most studies have focused on these constructs in isolation rather than examining their potential interactions (Brown and Ryan, 2003; Garland et al., 2017). Although some research has considered how mindfulness may moderate the relationship between personality traits and stress (Drake et al., 2017; Shapiro et al., 2011), few studies have explored both the moderating and mediating roles of specific mindfulness facets. Additionally, understanding how individuals’ experiences of stress and mindfulness practices align with quantitative findings could provide a more detailed picture of these processes. This study aims to fill these gaps by examining the independent, moderating, and mediating roles of mindfulness-attention and mindfulness-acceptance in the relationships between Five-Factor Model personality traits and perceived stress in Chinese adults. Using a mixed-methods design, this research combines quantitative analysis with qualitative exploration of participants’ experiences to uncover the mechanisms and contextual factors affecting stress perception and mindfulness practice.

## Literature review

### The role of personality traits in stress

Research indicates complex relationships between personality traits and perceived stress. The Five-Factor Model (FFM) of personality includes five dimensions: neuroticism, agreeableness, conscientiousness, extraversion, and openness to experience (Costa and McCrae, 1992; John and Srivastava, 1999; Soto and Jackson, 2013). These traits play a crucial role in how individuals perceive and cope with stress (McCrae and Costa, 1999). Neuroticism, associated with emotional instability and anxiety, is a consistent predictor of stress (Afshar et al., 2015; Kendler et al., 2006; Lahey, 2009; Ormel et al., 2013). Individuals with high neuroticism often experience higher stress levels and engage in maladaptive coping strategies like avoidance and rumination, which can exacerbate stress (Yoon et al., 2013). In contrast, agreeableness, which involves kindness and cooperation, is linked to lower perceived stress. Those high in agreeableness are more likely to use effective coping strategies, such as seeking social support and problem-solving, to manage stress (Graziano and Eisenberg, 1997; Saksvik and Hetland, 2011).

While neuroticism is generally viewed as a vulnerability factor for stress, other traits within the FFM framework—such as agreeableness, conscientiousness, extraversion, and openness to experience—are often associated with protective effects that can enhance resilience to stress and promote adaptive coping (Oshio et al., 2018). Conscientiousness, involving self-discipline and goal-directed behavior, offers protection against stress. Individuals with high conscientiousness manage stress effectively through proactive coping and planning (Luo et al., 2023; Roberts et al., 2009). Extraversion, characterized by sociability and assertiveness, is associated with lower stress levels, as extraverts often engage in social activities that buffer against stress (Eysenck, 1990; Watson and Clark, 1997). Openness to experience, marked by a willingness to explore new ideas, contributes to adaptive stress responses through cognitive flexibility and creativity in problem-solving (DeYoung, 2015; McCrae, 1996).

Neuroticism is consistently identified as a vulnerability factor for high stress across various contexts (Kendler et al., 2006; Luo et al., 2023; Piekarska, 2020). In contrast, extraversion, agreeableness, conscientiousness, and openness are generally linked to lower stress and more effective stress management (Baumann and Kuhl, 2005; Judge et al., 2002; Yan et al., 2024).

The protective nature of agreeableness is evident in its association with supportive social interactions and conflict resolution strategies that buffer against stress (Graziano and Eisenberg, 1997; Luo et al., 2023; McCrae and Sutin, 2009). Conscientious individuals often use proactive coping methods like time management and goal setting to mitigate stress (Roberts et al., 2009). Extraversion contributes to stress resilience through social engagement and positive affect, enhancing overall well-being (Sarubin et al., 2015; Watson and Clark, 1997). Openness fosters adaptive coping through cognitive flexibility and a readiness to explore alternative solutions (McCrae, 1996; Parker et al., 2015).

The relationship between personality traits and stress has been extensively studied, with research highlighting both protective and risk-enhancing roles that different traits play in stress responses (Connor-Smith and Flachsbart, 2007; Lecic-Tosevski et al., 2011; Luo et al., 2023). Neuroticism consistently emerges as a significant risk factor, closely tied to heightened emotional reactivity and tendencies toward negative affect and maladaptive coping strategies (Gashi et al., 2023; Leger et al., 2016). This trait predisposes individuals to experience greater stress, especially in situations perceived as challenging or uncontrollable, such as during the COVID-19 pandemic, where neuroticism was linked to increased emotional distress and reliance on passive coping (Afshar et al., 2015; Liu et al., 2021). These findings underscore neuroticism’s role in amplifying stress responses and hindering effective coping (Byrne et al., 2015; Gashi et al., 2023).

Conscientiousness, extraversion, and openness to experience, on the other hand, generally exhibit protective qualities against stress, promoting resilience and adaptive coping (Mammadov et al., 2024; Oshio et al., 2018). These traits are often associated with constructive stress management strategies, such as goal-directed behaviors, flexibility, and social engagement (Oshio et al., 2018; Hengartner et al., 2017). For instance, in academic and emergency settings, individuals high in conscientiousness and extraversion tend to demonstrate lower stress levels and more effective coping techniques, as they leverage planning, social support, and structured problem-solving to manage stress (Pollak et al., 2020; Saksvik and Hetland, 2011). Similarly, openness to experience, which fosters curiosity and cognitive flexibility, contributes to adaptive stress responses, enhancing resilience in dynamic or demanding contexts (Oshio et al., 2018).

Research further emphasizes the importance of contextual factors in shaping how personality traits influence stress perceptions and coping. In workplace and educational settings, traits like conscientiousness may interact with environmental demands, either buffering or intensifying stress responses depending on the context (Pollak et al., 2020). For example, conscientiousness promotes effective stress management in structured academic tasks requiring persistence and organization (Zhou et al., 2017). Conversely, neuroticism’s association with heightened stress can be particularly pronounced in high-pressure scenarios, where individuals may struggle with decision-making under stress, as observed by Byrne et al. (2015).

In summary, the Big Five personality traits influence stress management and coping differentially, depending on both the trait itself and the specific environmental context. Neuroticism heightens vulnerability to stress and maladaptive coping, while traits such as conscientiousness, extraversion, and openness foster resilience and adaptive strategies. These insights highlight the necessity of developing stress management interventions that consider both individual personality profiles and situational demands to bolster stress resilience effectively.

### Mindfulness and stress management

Mindfulness, with origins in ancient Buddhist practices, has gained prominence in psychological research for its role in improving mental health and well-being (Goldberg et al., 2018; Hathaisaard et al., 2022). As defined by Kabat-Zinn (2003), mindfulness involves purposeful and nonjudgmental attention to the present moment. It includes facets such as observing, describing, acting with awareness, non-judging, and non-reactivity (Baer et al., 2006; Zoogman et al., 2015). Observing involves noticing internal and external experiences like thoughts and sensations, while describing involves putting these experiences into words. Acting with awareness contrasts with automatic behavior, emphasizing focused engagement in activities. Non-judging involves adopting a neutral stance toward thoughts and feelings, and non-reactivity means allowing experiences to pass without being carried away by them (Baer et al., 2006; Bishop et al., 2004; Khoury et al., 2015).

Mindfulness practices aid in stress reduction and emotional regulation by fostering awareness and acceptance of the present moment (Fazia et al., 2020; Kabat-Zinn, 1990; Williams and Kabat-Zinn, 2013). By promoting observation of thoughts and emotions without judgment, mindfulness helps lessen the impact of stressors and encourages adaptive coping (Chiesa and Serretti, 2009; Sharma and Rush, 2014). Techniques like mindful breathing, body scans, and mindful movement (e.g., yoga) help individuals stay grounded during stressful situations (Rogers and Maytan, 2019; Zeidan et al., 2010). These practices may reduce physiological stress by lowering cortisol levels and enhancing parasympathetic nervous system activity (Pascoe et al., 2017). Mindfulness also plays a key role in emotional regulation by increasing awareness of emotional triggers and habitual reactions, allowing for more thoughtful responses rather than impulsive reactions (Guendelman et al., 2017; Hölzel et al., 2011; Teper et al., 2013; Zhang and Fathi, 2024). This process involves recognizing and accepting emotions as they arise, which can prevent the escalation of negative feelings and reduce the frequency of stress-related responses (Garland et al., 2017; Mohammad Hosseini et al., 2024).

Mindfulness-based interventions (MBIs), particularly mindfulness-based stress reduction (MBSR), are widely recognized for their effectiveness in managing stress and enhancing psychological well-being (Zhang et al., 2021). Developed by Kabat-Zinn (1990), MBSR integrates meditation, body scanning, and yoga as structured techniques for alleviating stress, anxiety, and pain (Grossman et al., 2004). Studies consistently show that MBSR reduces perceived stress, anxiety, and depression while enhancing overall mental health through cognitive flexibility and diminished negative thought patterns (Chiesa and Serretti, 2009; De Vibe et al., 2013; Fathi et al., 2023). Neuroimaging research further supports the impact of mindfulness on the brain, linking MBSR with changes in regions associated with attention, emotional regulation, and self-referential processing, including the prefrontal cortex, amygdala, and insula, which contribute to heightened resilience and emotional stability (Hölzel et al., 2011; Tang et al., 2015). Meta-analyses confirm MBSR’s broad applicability, showing it to be effective across clinical, educational, and occupational settings for reducing burnout, enhancing well-being, and supporting productivity (De Vibe et al., 2012; Grossman et al., 2004).

In healthcare settings, where professionals often face high stress and burnout, MBSR has demonstrated particular benefits. Studies indicate that MBSR significantly enhances mental health and lowers stress among healthcare providers, underscoring its relevance in demanding work environments (Bamber and Schneider, 2016; Irving et al., 2009). A systematic review by Kriakous et al. (2021) highlighted MBSR’s positive impact on healthcare workers by significantly improving psychological functioning and reducing symptoms of stress, anxiety, and burnout. The COVID-19 pandemic further illustrated MBSR’s utility; Marotta et al. (2022) found that MBSR effectively mitigated stress and burnout among Italian healthcare workers, suggesting it could enhance resilience during crises. Other research similarly supports MBSR’s role in preventing burnout and promoting well-being in high-stress professions, demonstrating its value as a mental health intervention for healthcare and related occupations (Molek-Winiarska and Żołnierczyk-Zreda, 2018; Zhang et al., 2024).

Beyond healthcare, MBSR has shown significant benefits in nonclinical settings, extending to students and athletes. Querstret et al. (2020) found in their meta-analysis that MBSR and mindfulness-based cognitive therapy (MBCT) enhance psychological health and well-being in general populations, supporting the broader applicability of mindfulness interventions. In athletic contexts, MBSR also contributes to mental resilience. For example, Jones et al. (2020) observed that MBSR improved psychological well-being, sleep quality, and performance among female collegiate rowers, suggesting its value in high-performance and physically demanding environments.

The reach of MBSR has been further extended through digital platforms, making mindfulness interventions more accessible (Sanilevici et al., 2021). Recent studies confirm the effectiveness of internet-based MBSR programs, especially in circumstances where in-person sessions are challenging, such as during the COVID-19 pandemic. Zhang et al.’s (2020) meta-analysis showed that online mindfulness interventions significantly reduced stress levels, suggesting that digital formats are scalable options for diverse populations. Sanilevici et al. (2021) also demonstrated the adaptability of MBSR to online formats, finding that a synchronous online MBSR program improved mental well-being and emotion regulation during the pandemic’s early stages. Likewise, research by Beer et al. (2020) and Yang et al. (2018) confirmed that mobile and online MBSR interventions reduced stress and enhanced health outcomes across various settings, emphasizing the flexibility of digital mindfulness approaches for stress management.

Taken together, these findings highlight MBSR as a highly effective, versatile intervention for stress management, capable of adapting to different populations and contexts. Its success across diverse settings and delivery methods reinforces its value as an accessible, adaptable approach to resilience-building, well-being enhancement, and stress reduction.

### The moderating and mediating role of mindfulness

Moderation involves a third variable that influences the strength or direction of the relationship between two variables. In this study, we hypothesize that mindfulness can modify the effect of personality traits on stress, potentially buffering the negative impact of traits like neuroticism. People high in neuroticism often experience higher stress levels due to tendencies toward negative emotions and poor stress management (Lahey, 2009; Metts et al., 2021). Mindfulness practices can reduce this effect by promoting better emotional regulation and cognitive flexibility (Garland et al., 2017). Feltman et al. (2009) found that mindfulness moderated the relationship between neuroticism and stress; individuals high in neuroticism reported lower stress levels when they had developed mindfulness skills.

Mindfulness can also enhance the positive effects of traits like conscientiousness and agreeableness (Banfi and Randall, 2022; Winning and Boag, 2015). Conscientious individuals already use effective coping strategies and self-regulation (Roberts et al., 2009), but mindfulness can further improve their focus on the present moment and self-discipline, reducing stress (Shapiro et al., 2006). Those high in agreeableness, who often use social support to cope, may find that mindfulness practices enhance their ability to engage empathetically and maintain harmonious relationships, thereby reducing stress (Baer et al., 2006; Graziano and Eisenberg, 1997; Nelson, 2014; Özer, 2022).

In addition, we propose that mindfulness may serve as a mediator in the relationship between personality traits and stress, explaining how personality traits influence stress levels. Mindfulness could mediate the impact of personality on stress by fostering adaptive coping mechanisms and enhancing emotional resilience (Hölzel et al., 2011; Keng et al., 2011). Neuroticism, for example, is often linked to heightened stress due to emotional instability (Ormel et al., 2013). Mindfulness may help mitigate this by enabling individuals to observe their thoughts and emotions without judgment, reducing stress escalation (Brown and Ryan, 2003). Bränström et al. (2011) found that mindfulness mediated the relationship between neuroticism and psychological distress, suggesting that mindfulness practices could help neurotic individuals manage stress more effectively.

Mindfulness can also mediate the relationship between other personality traits and stress. For example, extraverted individuals often experience lower stress due to their positive outlook and social engagement (Harris et al., 2017; Watson and Clark, 1997). Mindfulness can enhance these benefits by fostering present-moment awareness and reducing ruminative thinking, further decreasing stress (Tang et al., 2015; Zeidan et al., 2010). Ciesla et al. (2012) found that mindfulness mediated the relationship between conscientiousness and stress, indicating that mindfulness practices help conscientious individuals manage tasks with less stress. Similarly, Bowlin and Baer (2012) showed that mindfulness mediated the relationship between agreeableness and stress, highlighting how mindfulness enhances the stress-buffering effects of social support and empathy.

In summary, this study hypothesizes that mindfulness may both moderate and mediate the relationship between personality traits and perceived stress. By enhancing emotional regulation, cognitive flexibility, and present-moment awareness, mindfulness could buffer the adverse effects of neuroticism and reinforce the positive influences of traits like conscientiousness and agreeableness. This dual role of mindfulness—moderating stress vulnerability and mediating stress-reducing processes—suggests that mindfulness-based interventions may improve resilience and well-being across different personality profiles.

### Rationale for the current study

Although research on personality traits, mindfulness, and stress is extensive, gaps remain in understanding their combined effects. The Five-Factor Model (FFM) of personality is widely recognized for its role in predicting stress responses. Research suggests that mindfulness practices can reduce stress and improve emotional regulation (Kabat-Zinn, 2003; Grossman et al., 2004). However, it remains unclear how specific facets of mindfulness, such as mindfulness-attention and mindfulness-acceptance, may interact uniquely with different personality traits to influence perceived stress (Baer et al., 2006; Hölzel et al., 2011).

Most studies have examined personality traits and mindfulness separately rather than exploring their potential synergistic effects (Brown and Ryan, 2003; Garland et al., 2017). Furthermore, while some research has looked at the moderating role of mindfulness in the relationship between personality traits and stress, comprehensive studies examining both the moderating and mediating roles of specific mindfulness facets are scarce (Bränström et al., 2011; Feltman et al., 2009). This lack of exploration limits our understanding of how mindfulness can affect the stress process in more nuanced ways.

This study addresses these gaps by investigating the independent, moderating, and mediating roles of mindfulness-attention and mindfulness-acceptance in the relationships between FFM personality traits and perceived stress. Mindfulness-attention involves maintaining focus on present experiences, while mindfulness-acceptance refers to an open, non-judgmental attitude toward these experiences (Bishop et al., 2004; Cavanagh et al., 2014; Garland and 2017).

Focusing on these specific facets allows for a more detailed understanding of how mindfulness influences stress. The study will explore how mindfulness-attention and mindfulness-acceptance individually contribute to reducing perceived stress. It will also examine whether these mindfulness facets modify the relationship between personality traits and stress, potentially buffering negative effects associated with traits like neuroticism (Garland et al., 2017; Shapiro et al., 2006). Finally, it will investigate whether mindfulness-attention and mindfulness-acceptance explain how personality traits influence stress, offering insights into underlying mechanisms (Hölzel et al., 2011; Keng et al., 2011).

The study aims to integrate perspectives on personality and mindfulness to provide a more comprehensive understanding of stress management. The findings could guide the development of targeted mindfulness-based interventions tailored to individual personality profiles, potentially enhancing their effectiveness in reducing stress and improving well-being (Ciesla et al., 2012; Khoury et al., 2015).

To guide this exploration, the following hypotheses are proposed:

**H1:** Neuroticism will be positively associated with perceived stress, while conscientiousness, extraversion, agreeableness, and openness to experience will be negatively associated with perceived stress.

**H2:** Mindfulness-attention and mindfulness-acceptance will be negatively associated with perceived stress.

**H3:** Mindfulness-attention and mindfulness-acceptance will moderate the relationships between personality traits and perceived stress, such that the negative association between certain personality traits (e.g., neuroticism) and perceived stress will be weaker at higher levels of mindfulness.

**H4:** Mindfulness-attention and mindfulness-acceptance will mediate the relationships between personality traits and perceived stress, explaining how personality traits influence stress perception.

## Materials and methods

This study employed a sequential explanatory mixed-methods design to provide a comprehensive understanding of the relationships between personality traits, mindfulness, and perceived stress among Chinese adults. This approach is particularly suitable for exploring complex phenomena, as it allows for the collection of both quantitative and qualitative data (Creswell and Plano Clark, 2018). The mixed-methods approach involved an initial quantitative phase, followed by a qualitative phase aimed at elaborating and explaining the quantitative findings (Ivankova et al., 2006). This integration of methods enables a more nuanced understanding by supplementing statistical analysis with participants’ personal experiences and perspectives, offering a richer context for interpreting the results (Tashakkori and Teddlie, 2010). The primary purpose was to gain deeper insights into the quantitative results by exploring participants’ subjective experiences related to stress, mindfulness, and personality traits.

### Participants

Participants were recruited using convenience and snowball sampling methods. Convenience sampling involved sharing study information through online platforms (e.g., social media, online forums, university websites) and offline methods (e.g., flyers in community centers, libraries, and university campuses). Snowball sampling encouraged participants to share the study information with their networks. Inclusion criteria were: (a) ages 18 to 65, (b) fluency in Mandarin Chinese, and (c) ability to complete an online survey. Exclusion criteria were: (a) a history of severe mental health conditions (e.g., psychosis, bipolar disorder) and (b) current participation in a psychological intervention. Out of 850 individuals who showed interest, 637 met the criteria and completed the survey, resulting in a 75% response rate.

The final sample included 412 females (64.7%) and 225 males (35.3%), with an average age of 38.5 years (SD = 11.8). Efforts were made to ensure diversity in educational background, employment status, marital status, income, and geographic location within China.

After the quantitative analysis, a purposive sample of 26 participants was selected for the qualitative phase. This selection was based on specific criteria, such as high or low scores on perceived stress, mindfulness, or distinct personality traits. This approach aimed to capture a range of experiences related to the key variables from the quantitative analysis. The qualitative subset was chosen to ensure variation in demographics, including age, gender, and socioeconomic status, offering a more comprehensive understanding of how these factors manifest in daily life and coping strategies.

This study complied with the Declaration of Helsinki and was approved by the Institutional Review Board of Guizhou University of Finance and Economics. Prior to participation, individuals reviewed a Participant Information Sheet detailing the study’s purpose, procedures, potential risks and benefits, and their right to withdraw. Informed consent was obtained electronically, and participants were required to answer comprehension questions to ensure understanding. All data were anonymized and stored securely on a password-protected server. No personally identifiable information was collected. Data will be retained for 5 years and then securely destroyed. Participants were informed of the potential use of de-identified data for future research or publication.

### Measures

#### Demographics

Participants provided self-reported demographic information, including age, gender, educational level, employment status, marital status, and income level. This information was used to characterize the sample and to explore potential demographic influences on the relationships between personality, mindfulness, and perceived stress. Additionally, participants were asked to report their geographical location, allowing for an analysis of potential regional differences in stress perception and mindfulness practices across different areas in China.

#### Five-factor model personality traits

Personality traits were assessed using the Chinese version of the Big Five Inventory-2 (BFI-2; Soto and John, 2017; Zhang et al., 2022), which measures neuroticism, extraversion, agreeableness, conscientiousness, and openness to experience. The BFI-2 includes 60 items on a 5-point Likert scale (1 = disagree strongly to 5 = agree strongly), with higher scores indicating greater levels of each trait. Each trait is evaluated by 12 items, with three facets per trait, enhancing the assessment’s precision. Neuroticism includes anxiety, depression, and emotional volatility; extraversion encompasses sociability, assertiveness, and energy level; agreeableness assesses compassion, politeness, and trust; conscientiousness measures organization, productiveness, and responsibility; and openness captures intellectual curiosity, aesthetic sensitivity, and creativity. The CFA indicated acceptable model fit (CFI = 0.92, TLI = 0.91, RMSEA = 0.05, 90% CI [0.04, 0.06], SRMR = 0.04), supporting the construct validity of the BFI-2 in this context. Internal consistency was strong, with Cronbach’s alpha coefficients ranging from 0.79 to 0.88.

### Mindfulness

Mindfulness was assessed with the Chinese version of the Five Facet Mindfulness Questionnaire–Short Form (FFMQ-SF; Baer et al., 2006; Deng et al., 2011), a 15-item scale measuring observing, describing, acting with awareness, non-judging, and non-reactivity. Items were rated on a 5-point Likert scale from 1 (never or very rarely true) to 5 (very often or always true). Following prior research (Baer et al., 2006; Hölzel et al., 2011), we focused on two core facets: mindfulness-attention and mindfulness-acceptance. Mindfulness-attention was derived from the ‘observing’ and ‘acting with awareness’ subscales, while mindfulness-acceptance combined the ‘non-judging’ and ‘non-reactivity’ subscales. The ‘describing’ subscale was excluded, as it does not align with our study’s focus on attention and acceptance. The mindfulness-attention composite (6 items) showed good internal consistency (*α* = 0.81), and the mindfulness-acceptance composite (6 items) demonstrated similarly high reliability (α = 0.84). Confirmatory factor analysis (CFA) supported construct validity for the five-factor structure (CFI = 0.93, TLI = 0.91, RMSEA = 0.05, 90% CI [0.04, 0.06], SRMR = 0.05). The overall FFMQ-SF also showed acceptable reliability (α = 0.83), with subscale alphas ranging from 0.76 to 0.85.

### Perceived stress scale

Perceived stress was assessed using the Chinese version of the Perceived Stress Scale (PSS-10; Cohen et al., 1983; Leung et al., 2010). The scale includes 10 items on a 5-point Likert scale (0 = never to 4 = very often), with higher scores indicating greater perceived stress. The PSS-10 measures perceptions of life as unpredictable, uncontrollable, and overloaded, with items such as feeling unable to control important things and feeling nervous and stressed. The PSS-10 has established reliability and validity, including in Chinese samples (Leung et al., 2010). In this study, CFA indicated a strong model fit (CFI = 0.94, TLI = 0.92, RMSEA = 0.04, 90% CI [0.03, 0.06], SRMR = 0.03), affirming its reliability and validity. Cronbach’s alpha was 0.86.

### Semi-structured interviews

To supplement the quantitative findings, semi-structured interviews were conducted. An interview guide was developed to explore participants’ perceptions and experiences related to personality traits, mindfulness practices, and stress levels. Open-ended questions such as “Can you describe how you typically manage stress in your daily life?” and “How do you incorporate mindfulness into your routine, if at all?” were used to delve into the underlying mechanisms and contexts that could explain the quantitative results. The guide was pilot-tested to ensure clarity and relevance, with minor revisions made based on feedback. Interviews were conducted via video call, lasting 45–60 min, and were audio-recorded with consent for transcription and analysis. These interviews provided detailed insights into how personality and mindfulness influence stress experiences, offering a deeper understanding of the quantitative data.

### Procedure

The study was conducted from August 13 to October 12, 2023, using a sequential explanatory mixed-methods design. The participants were recruited through online platforms (WeChat, Weibo, mental health websites) and offline methods (flyers and posters in community centers and universities) with support from local mental health organizations. The eligible participants accessed a secure university-hosted survey platform, completed informed consent, and then answered a demographic questionnaire along with three measures: the Big Five Inventory-2 (BFI-2), Five Facet Mindfulness Questionnaire-Short Form (FFMQ-SF), and Perceived Stress Scale (PSS-10), presented in randomized order. Data security was maintained through encryption and restricted access. Following the quantitative phase, 26 participants participated in video-call interviews exploring stress management, mindfulness practices, and personality’s influence on coping.

### Data analysis

Data analysis for the quantitative phase was conducted using IBM SPSS Statistics (Version 28) and Hayes’ PROCESS macro (Model 4; Hayes, 2013). Preliminary analyses involved data screening to ensure quality and adherence to statistical assumptions. Missing data, which constituted less than 2% for any individual variable and were randomly distributed (Little’s MCAR test, *χ*^2^ = 25.63, df = 22, *p* = 0.27), were handled using pairwise deletion to maximize statistical power. This approach is deemed appropriate when the proportion of missing data is small and missingness is not systematically related to study variables (Enders, 2010).

Outliers were identified as cases with standardized z-scores on any continuous variable exceeding ±3.29. These extreme values were winsorized, meaning they were replaced with the next highest or lowest non-outlying value, to mitigate their undue influence on the analyses while preserving valuable data points (Field, 2013). Descriptive statistics, including means, standard deviations, and ranges, were calculated for all study variables. Bivariate Pearson correlations were computed to examine the interrelationships between personality traits, mindfulness facets, and perceived stress.

Hierarchical multiple linear regression analyses were employed to evaluate the independent and combined effects of personality traits and mindfulness on perceived stress. The initial model (Model 1) included the five personality traits (neuroticism, extraversion, agreeableness, conscientiousness, and openness) as predictors. In the second model (Model 2), mindfulness-attention and mindfulness-acceptance were added to assess their incremental contribution to explaining variance in perceived stress beyond personality traits.

To investigate potential moderation effects, Hayes’ PROCESS macro (Model 1) was used to test the interactions between each personality trait and each mindfulness facet in predicting perceived stress. When significant interactions were detected, simple slopes analyses were performed to probe the effect of the focal predictor (personality trait) on perceived stress at high (+1SD) and low (−1 SD) levels of the moderator (mindfulness facet). The Johnson-Neyman technique was employed to identify regions of significance, indicating the range of moderator values for which the conditional effects of the predictor on the outcome were statistically significant (Preacher et al., 2006). Mediation effects were also explored using PROCESS macro (Model 4) to assess whether mindfulness facets mediated the relationship between personality traits and perceived stress.

The qualitative data were analyzed using thematic analysis, following Braun and Clarke’s (2006) six-phase framework to identify key themes and patterns complementing the quantitative findings. Analysis began with a thorough reading of transcripts for data familiarization, which informed the initial coding based on recurring words, phrases, and ideas (Braun and Clarke, 2006; Nowell et al., 2017). Coding combined inductive methods for new insights with deductive approaches guided by themes identified in the quantitative phase (Boyatzis, 1998; Saldaña, 2016).

Following coding, these codes were organized into broader themes, visualized through a thematic map to illustrate their relationships (Braun and Clarke, 2006). Themes were then reviewed and refined, with comparisons made to coded extracts and the entire dataset for coherence and accuracy (Terry et al., 2017). Each theme was clearly defined and named to capture its essence (Clarke and Braun, 2018). NVivo software supported data organization and retrieval, aiding in the generation of a comprehensive report of identified themes (QSR International, 2020). The qualitative findings were integrated with quantitative results to deepen understanding of interactions between personality traits, mindfulness, and perceived stress, providing contextual insights into participants’ experiences (Frost et al., 2019; Vasileiou et al., 2018).

## Results

### Quantitative results

#### Descriptive statistics

Table 1 presents descriptive statistics and correlations for the study variables.

**Table 1:** Descriptive statistics and correlations.

Variable	Mean(SD)	1	2	3	4	5	6	7	8
1.Neuroticism	22.87 (5.92)	1	−0.28**	−0.15*	0.18*	−0.08	−0.32**	−0.35**	0.42**
2.Extraversion	31.25 (6.18)		1	0.05	−0.13*	0.31**	−0.21**	−0.26**	−0.14
3.Agreeableness	33.14 (5.21)			1	−0.17*	0.11	−0.15*	−0.18*	−0.17*
4.Conscientiousness	34.62 (5.53)				1	−0.14	−0.28**	−0.30**	0.38**
5.Openness	29.03 (5.87)					1	−0.25**	−0.27**	0.23**
6.Mindfulness-Attention	25.12 (5.31)						1	0.68**	−0.45**
7.Mindfulness-Acceptance	26.89 (4.95)							1	−0.48**
8.Perceived Stress	19.54 (6.83)								1

*p < 0.05, **p < 0.01.

As expected, neuroticism positively correlated with perceived stress (*r* = 0.42, *p* < 0.001), supporting its role as a stress vulnerability factor. Conscientiousness also showed a moderate positive correlation with perceived stress (*r* = 0.38, *p* < 0.001). Conversely, both mindfulness facets, attention and acceptance, were strongly and negatively correlated with perceived stress (*r* = −0.45 and *r* = −0.48, respectively, *p* < 0.001). Mindfulness-attention and mindfulness-acceptance were also highly correlated (*r* = 0.68, *p* < 0.001). Extraversion was not significantly correlated with perceived stress.

#### Hierarchical multiple regression

To disentangle the unique and combined effects of personality traits and mindfulness facets on perceived stress, a hierarchical multiple regression analysis was conducted. In Step 1, the five personality traits (neuroticism, extraversion, agreeableness, conscientiousness, and openness) were entered as predictors. This model was statistically significant, accounting for 22% of the variance in perceived stress (R^2^ = 0.22, *p* < 0.001, adjusted R^2^ = 0.21). Among the personality traits, neuroticism (*β* = 0.29, *p* < 0.001, sr^2^ = 0.08) and conscientiousness (*β* = 0.15, *p* < 0.01, sr^2^ = 0.02) emerged as significant positive predictors. This suggests that neuroticism and conscientiousness uniquely contribute 8 and 2% of the variance in perceived stress, respectively, indicating that individuals higher in these traits tended to report higher levels of perceived stress. Extraversion, agreeableness, and openness did not significantly predict perceived stress in this initial model (Table 2).

**Table 2:** Hierarchical multiple regression predicting perceived stress.

Model	Predictors	R^2^	ΔR^2^	*F*	df1	df2	*p*
1	Neuroticism, Extraversion, Agreeableness, Conscientiousness, Openness	0.22		35.11	5	631	<0.001
2	Mindfulness-Attention, Mindfulness-Acceptance	0.34	0.12***	45.28	2	629	<0.001

***p < 0.001.

In Step 2, mindfulness-attention and mindfulness-acceptance were added to the model. This expanded model accounted for a significantly greater proportion of variance in perceived stress (R^2^ = 0.34, *p* < 0.001, adjusted R^2^ = 0.33). The inclusion of mindfulness facets led to a substantial increase in explained variance [ΔR^2^ = 0.12, *F*(2, 629) = 45.28, *p* < 0.001]. This change in R^2^ was statistically significant, as confirmed by a hierarchical F-test comparing the two models, indicating that the addition of mindfulness significantly improved the model’s overall predictive power (*F* change = 45.28, *p* < 0.001).

In the full model (Model 2), neuroticism (*β* = 0.26, *p* < 0.001, sr^2^ = 0.07) and conscientiousness (β = 0.14, *p* < 0.05, sr^2^ = 0.02) remained significant positive predictors, while mindfulness-acceptance emerged as a significant negative predictor (*β* = −0.25, *p* < 0.001, sr^2^ = 0.06). The semi-partial correlations indicate that neuroticism, conscientiousness, and mindfulness-acceptance uniquely explain 7, 2, and 6% of the variance in perceived stress, respectively, highlighting their independent contributions to stress perception.

Interestingly, mindfulness-attention, while negatively correlated with perceived stress in the bivariate analysis (*r* = −0.45, *p* < 0.001), was no longer a significant predictor in the full model. This suggests that the effect of mindfulness-attention on perceived stress is largely accounted for by its overlap with mindfulness-acceptance and the other personality traits in the model, particularly neuroticism. The shared variance between these variables reduces the unique explanatory power of mindfulness-attention in this context. Nevertheless, the overall contribution of the mindfulness facets to the model remains substantial, underscoring their collective importance in understanding stress perception.

#### Moderation analyses

To investigate the potential moderating effects of mindfulness on the relationship between personality traits and perceived stress, we conducted a series of moderation analyses using Hayes’ PROCESS macro (Model 1; Hayes, 2013). This approach allowed us to examine whether the strength or direction of the association between each personality trait and perceived stress varied depending on the level of mindfulness-attention or mindfulness-acceptance.

Contrary to our hypothesis, neither mindfulness-attention (*β* = 0.03, *p* = 0.52) nor mindfulness-acceptance (*β* = −0.02, *p* = 0.68) significantly moderated the relationship between neuroticism and perceived stress, indicating that the association between neuroticism and perceived stress does not vary by an individual’s level of mindfulness.

A significant interaction emerged between agreeableness and mindfulness-attention (*β* = −0.08, *p* = 0.03), suggesting a moderation effect. Simple slopes analyses indicated that the negative association between agreeableness and perceived stress was stronger for individuals with low mindfulness-attention (simple slope = −0.16, *p* < 0.001) compared to those with high mindfulness-attention (simple slope = −0.04, *p* = 0.12). No significant moderation effects were found for other personality traits interacting with either mindfulness-attention or mindfulness-acceptance.

#### Mediation analyses

To elucidate the pathways through which personality traits and mindfulness facets influence perceived stress, mediation analyses were conducted using Hayes’ PROCESS macro (Model 4; Hayes, 2013). Specifically, we tested whether mindfulness-attention and mindfulness-acceptance mediated the relationships between neuroticism and agreeableness with perceived stress. The results revealed a nuanced pattern of mediation effects.

For neuroticism, the total effect on perceived stress was significant (*b* = 0.42, SE = 0.04, *p* < 0.001). However, neither mindfulness-attention nor mindfulness-acceptance emerged as significant mediators of this association. The indirect effect of neuroticism on perceived stress through mindfulness-attention was non-significant (*b* = −0.01, 95% CI [−0.04, 0.02]), accounting for only 2.4% of the total effect. Similarly, the indirect effect through mindfulness-acceptance was also non-significant (*b* = −0.03, 95% CI [−0.08, 0.02]), explaining 7.1% of the total effect. This indicates that the effect of neuroticism on perceived stress is largely direct, rather than mediated by these mindfulness facets.

The total effect of agreeableness on perceived stress was significant (*b* = −0.17, SE = 0.04, *p* < 0.001). For agreeableness, a significant indirect effect was observed through mindfulness-acceptance (b = −0.06, 95% CI [−0.10, −0.02]), accounting for 35.3% of the total effect. This suggests that over one-third of the association between higher agreeableness and lower perceived stress is explained by increased mindfulness-acceptance. In contrast, the indirect effect through mindfulness-attention was not significant (*b* = −0.02, 95% CI [−0.06, 0.02]), indicating that mindfulness-attention does not play a substantial mediating role in this relationship.

These results highlight the differential roles of mindfulness facets in the stress-buffering process. While mindfulness-attention does not significantly mediate the relationships between personality and perceived stress, mindfulness-acceptance plays a substantial role in explaining the association between agreeableness and reduced stress levels (Table 3).

**Table 3:** Mediation analyses results.

Pathway	Total Effect (b)	Indirect Effect (b)	95% CI	% Mediated
Neuroticism → Mindfulness-Attention → Perceived Stress	0.42***	−0.01	[−0.04, 0.02]	2.4%
Neuroticism → Mindfulness-Acceptance → Perceived Stress	0.42***	−0.03	[−0.08, 0.02]	7.1%
Agreeableness → Mindfulness-Attention → Perceived Stress	−0.17***	−0.02	[−0.06, 0.02]	11.8%
Agreeableness → Mindfulness-Acceptance → Perceived Stress	−0.17***	−0.06*	[−0.10, −0.02]	35.3%

*p < 0.05, **p < 0.01, ***p < 0.001.

### Qualitative results

The qualitative phase of the study aimed to provide a deeper understanding of the quantitative findings by exploring participants’ lived experiences and perspectives regarding the relationship between their personality traits, mindfulness practices, and perceived stress. Through thematic analysis, three primary themes emerged from the semi-structured interviews: (1) Personality-Driven Stress Responses, (2) Mindfulness as a Moderating Mechanism, and (3) The Dynamic Interaction between Personality and Mindfulness.

#### Personality-driven stress responses

Participants’ responses to stress were distinctly shaped by their personality traits, aligning with the quantitative findings. Those scoring high in neuroticism exhibited a pronounced tendency to ruminate and experience heightened emotional reactivity, which often perpetuated a cycle of stress. Participant 8, who displayed high neuroticism, described this phenomenon vividly: “I often find myself overthinking even the smallest problems. It’s like a never-ending loop where I anticipate the worst possible outcomes, which in turn makes me more anxious. Even when there’s nothing immediate to worry about, I feel this underlying tension that something might go wrong.” This quote underscores the persistent anxiety that neurotic individuals face, illustrating how their anticipatory stress contributes to a chronic state of unease.

In contrast, participants characterized by higher levels of conscientiousness generally adopted proactive stress management strategies. They emphasized the role of organization and planning in mitigating stress. Participant 15, high in conscientiousness, shared: “When I feel overwhelmed, my first instinct is to make a list and tackle things one step at a time. I find that having a clear plan helps me feel more in control and reduces my stress. It’s like, if I have a strategy, then the problem does not seem as daunting.” This account highlights the efficacy of structured approaches in alleviating stress among conscientious individuals.

On the other hand, participants high in agreeableness often sought social support to buffer against stress but also reported the stress associated with prioritizing others’ needs over their own. Participant 22, who exhibited high agreeableness, noted: “I hate conflicts and always try to make sure everyone around me is happy. But sometimes, I end up taking on too much just to avoid saying no, and that stresses me out. It’s like I’m caught between wanting to help others and needing time for myself.” This statement reflects the internal conflict and stress that arise from the desire to maintain harmony while neglecting personal needs.

#### Mindfulness as a moderating mechanism

The role of mindfulness as a moderating factor in stress responses emerged strongly from the data, supporting the quantitative findings that mindfulness significantly moderates stress. Participants who actively engaged in mindfulness practices reported a greater ability to detach from stress-inducing thoughts and emotions. Participant 5, who scored high in mindfulness-attention, explained: “Mindfulness has taught me to observe my thoughts without getting caught up in them. When I start to feel overwhelmed, I take a moment to focus on my breathing and just notice what I’m feeling. This helps me to step back and not react impulsively. It’s not that the stress goes away, but I feel more grounded and less controlled by it.” This quote demonstrates the grounding effect of mindfulness on participants’ stress experiences.

Participants high in agreeableness, when coupled with mindfulness, found that mindfulness practices facilitated a more balanced approach to managing interpersonal stressors. Participant 12, high in both agreeableness and mindfulness-attention, commented: “I used to take on everyone’s problems as my own, but mindfulness has helped me realize that it’s okay to say no. I can be there for others without losing myself in their issues. It’s like I can be present and supportive, but also maintain my own space.” This insight reveals how mindfulness supports maintaining personal boundaries while managing social harmony.

For those high in mindfulness-acceptance, the ability to accept stressful situations without judgment was particularly pronounced. Participant 18, who demonstrated high mindfulness-acceptance, described: “Mindfulness has taught me that it’s okay to feel stressed or anxious. I used to beat myself up for feeling this way, but now I try to just sit with the discomfort and accept it as part of my experience. It does not make the stress go away, but it makes it more manageable because I’m not adding an extra layer of judgment on top of it.” This perspective illustrates how mindfulness-acceptance helps participants manage stress by reducing self-criticism and emotional resistance.

#### The dynamic interaction between personality and mindfulness

The interaction between personality traits and mindfulness practices was pivotal in shaping participants’ stress experiences. Participants high in openness to experience were notably more inclined to explore and integrate diverse mindfulness practices into their routines. Participant 3, who exhibited high openness, shared: “I’ve tried different mindfulness practices, like meditation, yoga, and even mindful walking. I think being open to new experiences has helped me find what works best for me in different situations. It’s not a one-size-fits-all thing; it’s about being curious and willing to experiment.” This statement highlights how openness fosters a flexible and adaptive approach to stress management.

Conversely, participants with lower levels of conscientiousness often struggled with maintaining a consistent mindfulness practice, though they acknowledged its benefits when practiced sporadically. Participant 19, low in conscientiousness, remarked: “I struggle with routines in general, so sticking to a regular mindfulness practice is hard for me. But when I do remember to take a moment and just be present, it really makes a difference. It’s just the consistency that I find challenging.” This quote illustrates the challenges faced by those with lower conscientiousness in integrating mindfulness into their routines.

Extraverted individuals reported that mindfulness enhanced their social interactions, leading to a greater sense of connection and reduced stress in social settings. Participant 10, high in extraversion and mindfulness, observed: “Mindfulness helps me to really be present when I’m with others. I feel more connected and less distracted by my own thoughts or worries. It’s like I can fully engage in the moment, which makes social interactions more enjoyable and less draining.” This account demonstrates how mindfulness can enhance social engagement and reduce stress for extraverted individuals.

Overall, the qualitative findings offer detailed insights into how personality traits and mindfulness practices interact to influence stress experiences. The data suggest that stress responses are affected by individual differences in traits like neuroticism, conscientiousness, and agreeableness. Mindfulness serves as a key moderating factor, helping participants manage stress through non-reactivity, acceptance, and better handling of interpersonal stressors.

## Discussion

This study examined the intricate relationships between personality traits, mindfulness facets, and perceived stress in a diverse sample of Chinese adults. Using a sequential explanatory mixed-methods design, we sought to unravel not only the independent effects of these factors, but also their potential interactions in shaping stress experiences. The findings offer a nuanced understanding of how dispositional and state factors converge to influence stress perception and coping.

### Personality traits and stress vulnerability

Hypothesis 1, which posited that neuroticism would be positively associated with perceived stress and that conscientiousness, extraversion, agreeableness, and openness would be negatively associated with stress, was partially supported. Neuroticism was a strong predictor of perceived stress, consistent with prior research identifying it as a vulnerability factor (Costa and McCrae, 1992; Lahey, 2009; Ormel et al., 2013). Individuals high in neuroticism are predisposed to negative emotionality and anxiety, which makes them more likely to interpret situations as threatening, thus engaging in maladaptive coping mechanisms that reinforce a cycle of stress (Barlow et al., 2021; Eysenck, 1990; Kendler et al., 2006; Paulus et al., 2016). This pattern was also reflected in the qualitative findings, where participants with high neuroticism reported a persistent sensitivity to daily stressors. For instance, minor setbacks often triggered a cascade of worry, underscoring the association between neuroticism, heightened physiological reactivity, and a chronic sense of unease (Lahey, 2009; Ormel et al., 2013). These insights provide a more nuanced view of how neurotic individuals perceive and experience stress as an almost constant aspect of their lives.

The positive correlation between conscientiousness and perceived stress deviates from some existing literature, necessitating a closer examination of how this trait influences stress perception (Judge et al., 2002; Soto and John, 2017). While conscientiousness is typically linked to effective stress management through proactive coping and organizational skills (Bogg and Roberts, 2004; Roberts et al., 2009), the current findings suggest a more complex relationship. Highly conscientious individuals might experience stress due to perfectionistic tendencies and a propensity for overcommitment (Besser and Shackelford, 2007; Stoeber and Otto, 2006). Qualitative data further clarified this dynamic, indicating that while conscientious participants used their organizational skills as a stress management strategy, they also felt significant internal pressure. The desire for perfection and the struggle to meet high personal standards contributed to their stress, particularly when time and resources were limited (Hill et al., 2010; Rice and Richardson, 2014). This pattern aligns with findings from Lin et al. (2015), who describe conscientiousness as a “double-edged sword” that can enhance both performance and stress reactions in high-stress contexts. Such results highlight that conscientiousness may heighten stress responses, particularly in individuals driven by achievement-oriented perfectionism, thereby intensifying the stress reaction when facing pressure or deadlines.

This nuanced view of conscientiousness and stress aligns with Murphy et al. (2013), who found that conscientiousness, while generally protective, could increase stress reactivity when individuals experience high levels of interpersonal stress. Furthermore, the work by Chen et al. (2022) suggests that conscientious individuals often benefit from a “stress-is-a-challenge” mindset that promotes adaptive coping; however, when they perceive stress as a threat, this mindset may fail, potentially leading to increased stress perception in perfectionistic or high-demand settings.

The absence of significant findings for extraversion, agreeableness, and openness to experience suggests the need for further investigation. Extraversion is generally associated with stress-buffering effects through social engagement and positive affect; however, this relationship may depend on specific contextual factors that were not fully captured in this study (Brown and Ryan, 2003; Eysenck, 1990; Watson and Clark, 1997). The qualitative data offered additional context, revealing that while extraverted participants often relied on social networks for stress relief, a lack of social opportunities, such as during periods of isolation, diminished their usual resilience. This indicates that the protective role of extraversion against stress may vary depending on the availability of social interactions (John and Srivastava, 1999; Soto and Jackson, 2013). Similarly, although agreeableness is often linked to lower stress through supportive social interactions, the qualitative findings indicated that agreeable individuals might experience internal conflict when managing interpersonal stress. In challenging social situations, the desire to maintain harmony often became a source of stress, especially when it conflicted with their own needs or expectations (Graziano and Eisenberg, 1997; McCrae and Sutin, 2009; Roberts et al., 2009).

Openness to experience and its relationship with stress perception present a complex picture. Openness, characterized by cognitive flexibility and a willingness to explore new experiences, can facilitate adaptive coping and effective problem-solving in stressful situations (DeYoung, 2015; McCrae, 1996; Zeidan et al., 2010). However, qualitative findings suggested that high openness could also lead to stress in environments that restrict creativity and exploration. For example, individuals with high openness reported feeling stressed in rigid or monotonous settings where they were unable to express their ideas freely. This underscores the importance of environmental fit in moderating the impact of openness on stress perception, suggesting that while individuals high in openness may thrive in dynamic and stimulating contexts, they may struggle in more restrictive environments (Kaufman et al., 2016; Silvia et al., 2011). This notion is also supported by Luo et al. (2023), who highlight the complex role of openness in different stress contexts, with openness potentially increasing stress perception in situations where flexibility and innovation are constrained.

### Mindfulness as a stress-buffering mechanism

Hypothesis 2 posited that both mindfulness-attention and mindfulness-acceptance would correlate negatively with perceived stress, a hypothesis that was strongly supported by our findings. Both facets of mindfulness, attention and acceptance, showed significant negative correlations with perceived stress, indicating the role of mindfulness in reducing stress levels (Baer et al., 2006; Brown and Ryan, 2003; Garland et al., 2017). However, the regression analyses identified mindfulness-acceptance as the more significant predictor. This suggests that the ability to accept and engage non-judgmentally with present experiences plays a more crucial role in mitigating stress than simply paying attention to the present moment (Hölzel et al., 2011; Keng et al., 2011). The qualitative data provided additional context, as participants described using mindfulness-acceptance in daily situations to manage overwhelming emotions. They emphasized the importance of acceptance over resistance, indicating that this approach made a noticeable difference in how they coped with stress. This aligns with research suggesting that mindfulness-acceptance facilitates emotional regulation and adaptive coping strategies (Garland et al., 2017; Hölzel et al., 2011). Neuroimaging studies reinforce these findings by showing increased activity in brain regions associated with emotion regulation and decreased activity in areas linked to stress and anxiety when engaging in mindfulness practices (Tang et al., 2015; Zeidan et al., 2010).

The qualitative data also highlighted distinct roles for each mindfulness facet. Participants frequently mentioned mindfulness-acceptance as a key strategy for managing stress, while the role of mindfulness-attention appeared more complex. Some participants indicated that focusing solely on the present moment without an attitude of acceptance could amplify their awareness of stressors, leading to heightened stress levels. This suggests that attention alone may not be as effective in reducing stress unless paired with an accepting mindset (Brown and Ryan, 2003; Hölzel et al., 2011). These observations underscore the importance of a balanced approach to mindfulness practice, where cultivating both attention and acceptance can enhance stress resilience (Baer et al., 2006; Garland et al., 2017).

### Moderating and mediating effects of mindfulness

Regarding hypothesis 3, which proposed that mindfulness facets would moderate the relationship between personality traits and stress, results indicated limited moderation effects. Specifically, mindfulness-attention moderated the relationship between agreeableness and perceived stress. This suggests that the stress-buffering effect of agreeableness is more pronounced when individuals are less attentive to their immediate experiences (Ciesla et al., 2012; Feltman et al., 2009). The qualitative data added depth to this finding, as agreeable participants reported that mindfulness practices helped them set healthier boundaries and manage interpersonal stress more effectively. They expressed that mindfulness enhanced their self-awareness, enabling them to recognize when they were overextending themselves and allowing them to set limits without experiencing guilt. This indicates that mindfulness contributes to emotional regulation in social interactions, reinforcing the stress-buffering effects associated with agreeableness (Kabat-Zinn, 2003; Shapiro et al., 2006).

Finally, hypothesis 4 suggested that mindfulness-attention and mindfulness-acceptance would mediate the relationship between personality traits and stress. Our mediation analyses revealed that mindfulness-acceptance partially mediated the relationship between agreeableness and stress, supporting the notion that acceptance facilitates adaptive coping. Individuals higher in agreeableness likely benefit from acceptance-based practices, which reduce their reactivity to stressors and foster emotional resilience (Grossman et al., 2004; Khoury et al., 2015).Individuals with higher levels of agreeableness might naturally gravitate towards acceptance-based coping strategies, and mindfulness practices can enhance this tendency. The qualitative data indicated that participants who were typically inclined to avoid conflict found mindfulness helpful in accepting situations that were not ideal. They recognized that it was not always necessary to please everyone, which aligns with the notion that acceptance helps reduce reactivity to stressors, thereby lowering overall stress perception (Garland et al., 2017; Keng et al., 2011).

### The dynamic interplay of personality and mindfulness

The qualitative data provided rich insights into the dynamic interaction between personality and mindfulness, revealing that the effectiveness of mindfulness practices may depend on an individual’s personality profile (DeYoung, 2015; McCrae and Costa, 1999). Individuals high in openness to experience displayed a greater inclination to explore and integrate diverse mindfulness practices, reflecting their inherent curiosity and willingness to embrace new experiences (McCrae, 1996; John and Srivastava, 1999). This suggests that tailoring mindfulness interventions to individual preferences and personality styles may enhance engagement and effectiveness, as openness is associated with a greater flexibility in coping strategies (Williams and Kabat-Zinn, 2013; Zeidan et al., 2010).

Conversely, individuals with lower conscientiousness reported challenges in maintaining consistent mindfulness practices, highlighting the potential influence of personality on adherence to mindfulness training (Roberts et al., 2009; Soto and Jackson, 2013). This underscores the importance of considering individual differences in self-discipline and motivation when designing and implementing mindfulness interventions (Baumann and Kuhl, 2005; van den Hurk et al., 2011). Future research could explore strategies to enhance engagement and adherence among individuals with varying personality profiles, potentially through personalized interventions that account for differences in motivation and self-regulation (Teper et al., 2013; Winning and Boag, 2015).

## Conclusion

This study clarifies the connections between personality traits, mindfulness, and perceived stress using a mixed-methods approach. Quantitative findings affirm that neuroticism predicts higher stress levels, underscoring its role as a vulnerability factor and the need to address emotional instability and negative affect in stress management. Conscientiousness and agreeableness were found to support stress management through proactive coping and social support. The qualitative phase provided further insights, showing how personality traits and mindfulness influence individual stress responses. High-neuroticism participants reported rumination and heightened emotional reactivity, while those high in conscientiousness highlighted organization and planning as key coping strategies. Agreeableness emerged as both a stress buffer in positive social interactions and a source of stress when participants prioritized others’ needs above their own, adding complexity to its role. Mindfulness-acceptance stood out as critical for stress resilience, helping individuals balance stress experiences with non-judgmental acceptance. Participants practicing mindfulness-acceptance reported an increased ability to distance themselves from stress-inducing thoughts, which strengthened their coping mechanisms. This capacity to “sit with discomfort” reduced the intensity of stress, demonstrating the unique benefits of mindfulness-acceptance in managing stress.

## Implications

The findings of this study have significant implications for developing and implementing stress management interventions, emphasizing the need for both mindfulness-based practices and strategies that consider individual personality traits. The key role of mindfulness-acceptance in stress reduction suggests that interventions such as Mindfulness-Based Stress Reduction (MBSR) and Acceptance and Commitment Therapy (ACT), which focus on cultivating acceptance, could be particularly effective. These programs can empower individuals to develop a non-judgmental attitude towards their experiences, fostering adaptive stress responses and improving well-being (Kabat-Zinn, 1990; Khoury et al., 2015). Qualitative insights further support this, highlighting how participants effectively used mindfulness-acceptance practices to manage stress by reducing emotional reactivity and maintaining a balanced engagement with stressors.

Additionally, the study’s qualitative insights underscore the importance of integrating personality traits into stress management approaches. For individuals high in neuroticism, interventions focusing on enhancing emotional regulation and reducing negative thought patterns may be particularly beneficial. Cognitive-behavioral therapy (CBT) could be a suitable approach for this group, as it equips individuals with strategies to challenge maladaptive thoughts and promote healthier coping mechanisms (Hofmann et al., 2012). The interviews further revealed how individuals high in neuroticism often experience a cycle of negative thinking, suggesting that targeted CBT techniques could help disrupt this pattern and encourage more adaptive stress responses.

For those high in conscientiousness, the study highlights the need for interventions that balance goal-directed behavior with relaxation and self-care. Qualitative data revealed that these individuals often experience stress due to perfectionism and overcommitment. Interventions promoting self-compassion, time management, and relaxation techniques could help them manage responsibilities more effectively and reduce burnout risk. Furthermore, the findings suggest that interventions should consider how personality traits interact with mindfulness practices. For example, mindfulness practices that emphasize boundary-setting and self-compassion could be beneficial for agreeable individuals, helping them maintain supportive relationships without compromising their own well-being.

## Limitations and future directions

Although this study provides important insights, several limitations need to be acknowledged, which also point to areas for future research. First, the use of self-report measures for personality traits, mindfulness, and perceived stress may introduce bias, as participants could underreport or overreport their experiences due to social desirability or recall errors. To address this, future research could incorporate objective measures, such as behavioral observations or physiological assessments, to supplement self-report data and improve the validity of the findings. Second, the cross-sectional design limits the ability to establish causal relationships among the variables. Although the results suggest possible causal pathways, longitudinal studies are needed to confirm the temporal dynamics and directional influences between personality, mindfulness, and stress. Such studies could track changes in these variables over time and investigate how they interact to predict stress trajectories.

Third, although there was a gender imbalance in the sample, with predominantly female participants, the distribution was more balanced than typically observed in stress research samples. However, this imbalance still restricts the generalizability of findings to males. Future studies should aim for a more balanced gender representation to explore potential gender differences in the relationships between personality, mindfulness, and stress, enhancing the research’s applicability and external validity. Fourth, the study utilized convenience sampling methods, which, while effective for recruiting participants, limit the generalizability of the findings. Future research using probability sampling methods could improve the representativeness of the sample and enhance the robustness of the conclusions.

Fifth, while the qualitative data provided valuable context for the quantitative findings, it mainly focused on participants’ subjective experiences. Future research could incorporate additional qualitative approaches, such as focus groups or diary studies, to capture the dynamic and contextual nature of stress experiences. This might help reveal how personality traits and mindfulness practices interact in real-time responses to specific stressors. Lastly, this study concentrated on a specific cultural context (Chinese adults). Future research should examine the generalizability of the findings to other populations and cultures. Investigating potential cultural differences in personality-mindfulness interactions and stress responses could contribute to a more nuanced understanding of these relationships.

## Data Availability

The raw data supporting the conclusions of this article will be made available by the authors, without undue reservation.
